# Supplementary material for: An alternative peptone preparation using Hermetia illucens (Black soldier fly) hydrolysis: process optimization and performance evaluation
Source: PeerJ. 2024 Feb 26;12:e16995. doi: 10.7717/peerj.16995 (PMC10903346; doi:10.7717/peerj.16995)
Supplement: File S1 — The pages 1 and 2 show the protease on HIL proteins at different temperatures additions time pH, and the page 3 shows the optimum inoculum of E. coli in HIL peptones and commercial peptones. [file peerj-12-16995-s002.pdf]

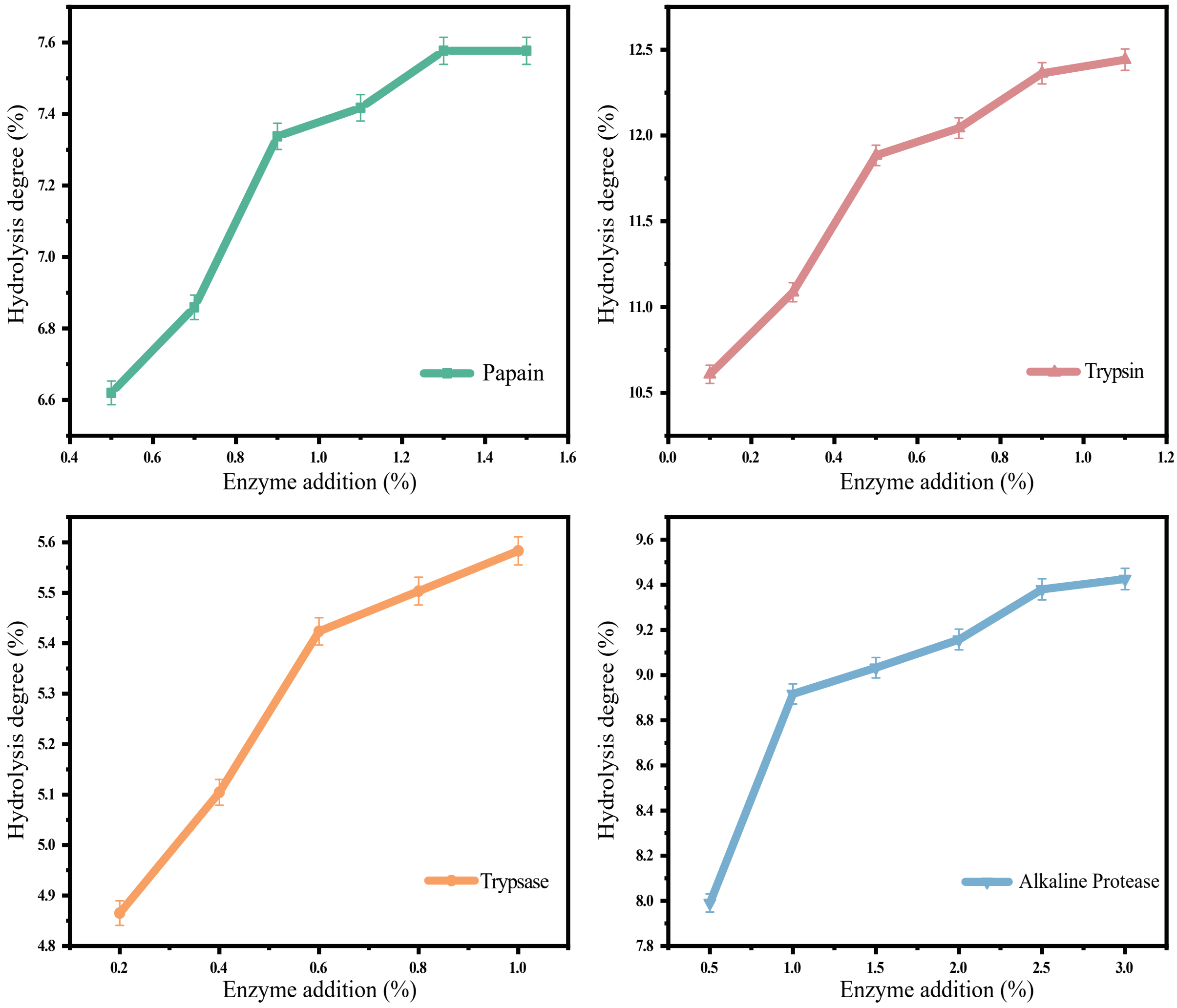

Effect of enzyme addition on the degree of hydrolysis of four enzymes

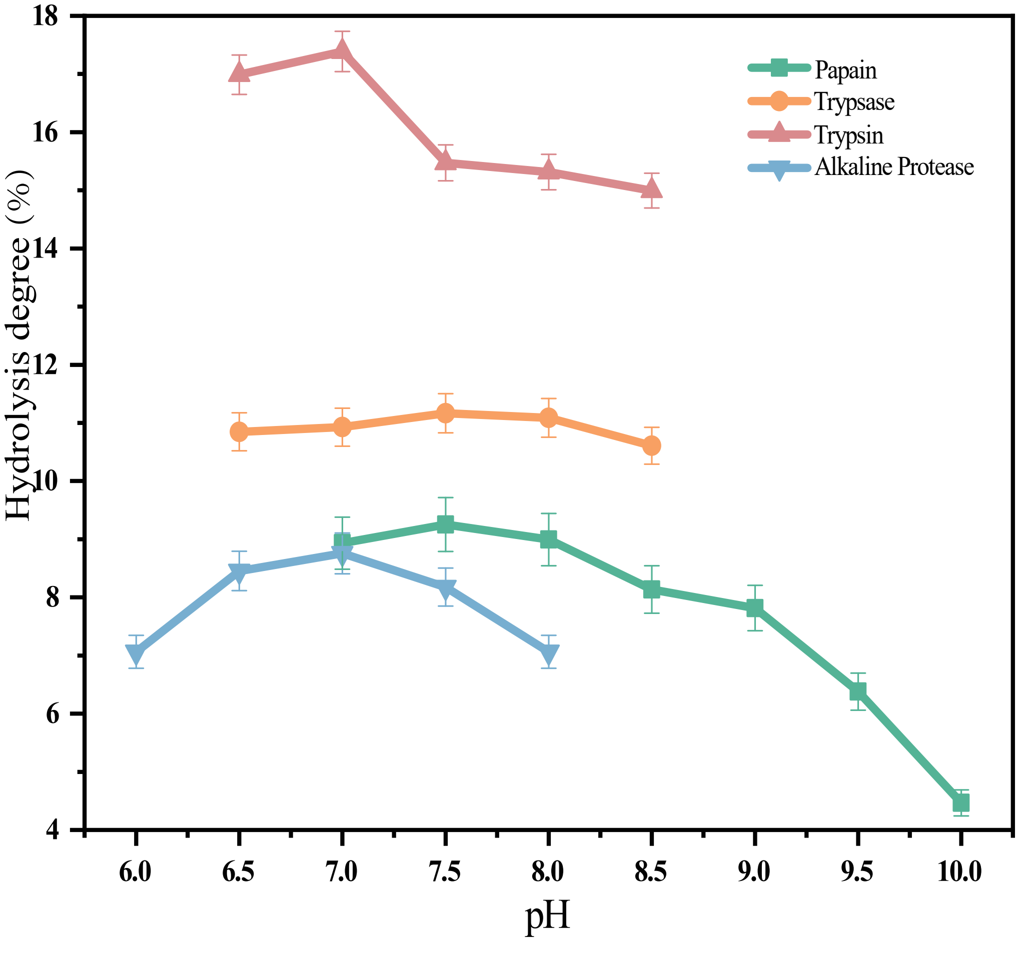

Effect of pH on the degree of hydrolysis of four enzymes

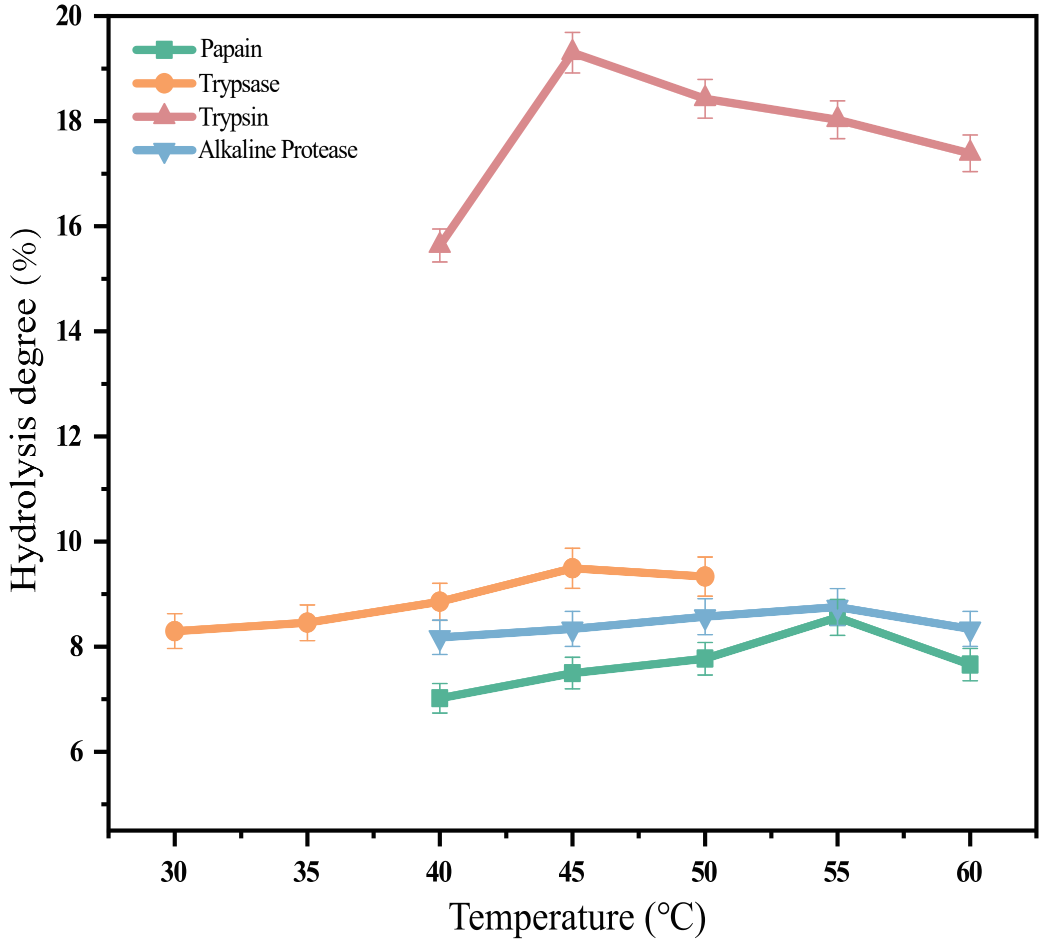

Effect of temperature on the degree of hydrolysis of four enzymes

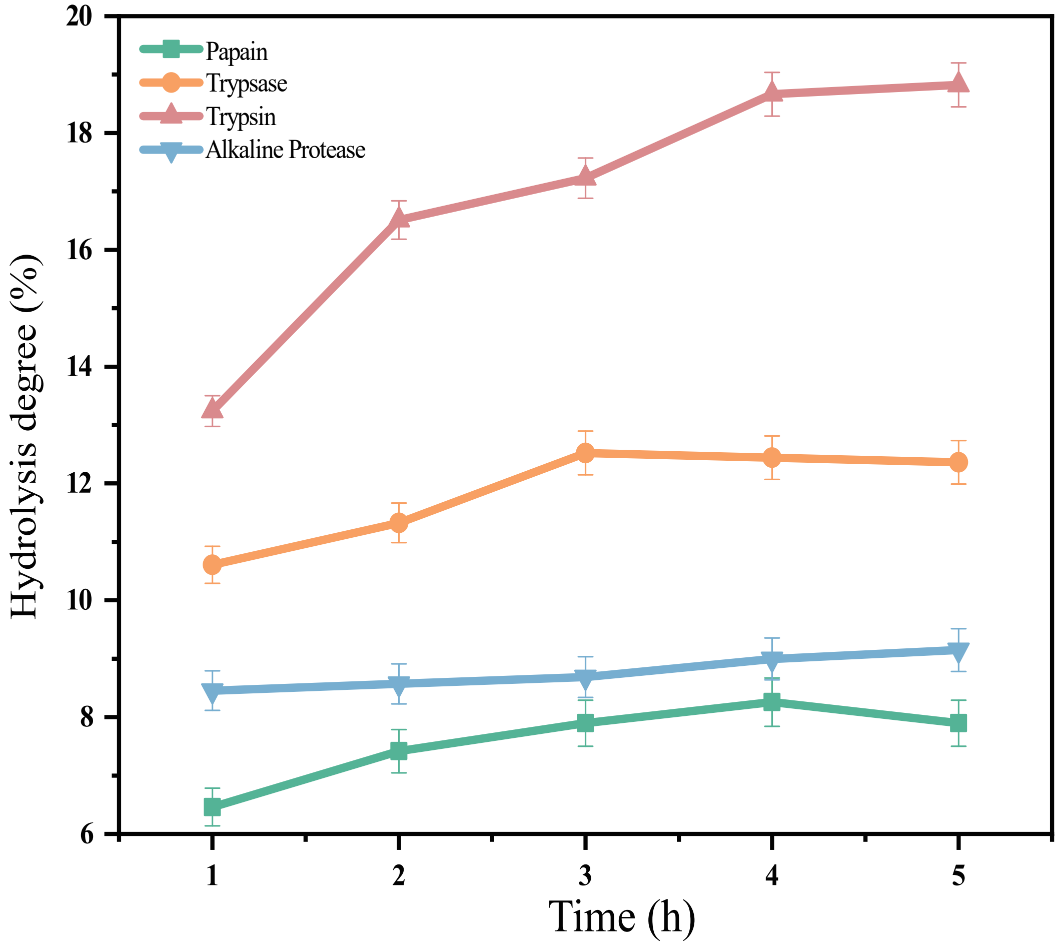

Effect of time on the degree of hydrolysis of four enzymes

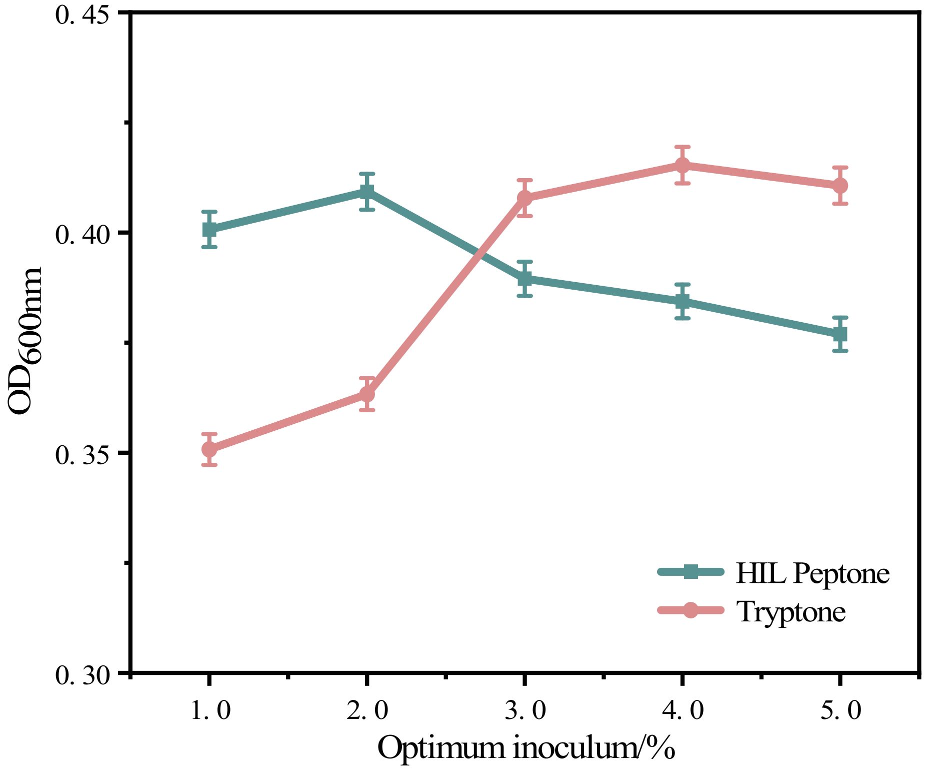

Determination of optimal inoculum size of *Escherichia coli*
